# Reciprocal Relationships between Trajectories of Depressive Symptoms and Screen Media Use during Adolescence

**DOI:** 10.1007/s10964-018-0901-y

**Published:** 2018-07-25

**Authors:** Stephen Houghton, David Lawrence, Simon C. Hunter, Michael Rosenberg, Corinne Zadow, Lisa Wood, Trevor Shilton

**Affiliations:** 1 Graduate School of Education, The University of Western Australia, Perth, WA Australia; 2 School of Psychological Sciences and Health, University of Strathclyde, Glasgow, Scotland; 3 Health Promotion Evaluation Unit, The University of Western Australia, Perth, WA Australia; 4 School of Population Health, The University of Western Australia, Perth, WA Australia; 5 National Heart Foundation, The University of Western Australia, Perth, WA Australia

**Keywords:** Depressive symptoms, Adolescence, Trajectories, Screen media use

## Abstract

Adolescents are constantly connected with each other and the digital landscape through a myriad of screen media devices. Unprecedented access to the wider world and hence a variety of activities, particularly since the introduction of mobile technology, has given rise to questions regarding the impact of this changing media environment on the mental health of young people. Depressive symptoms are one of the most common disabling health issues in adolescence and although research has examined associations between screen use and symptoms of depression, longitudinal investigations are rare and fewer still consider trajectories of change in symptoms. Given the plethora of devices and normalisation of their use, understanding potential longitudinal associations with mental health is crucial. A sample of 1,749 (47% female) adolescents (10–17 years) participated in six waves of data collection over two years. Symptoms of depression, time spent on screens, and on separate screen activities (social networking, gaming, web browsing, TV/passive) were self-reported. Latent growth curve modelling revealed three trajectories of depressive symptoms (low-stable, high-decreasing, and low-increasing) and there were important differences across these groups on screen use. Some small, positive associations were evident between depressive symptoms and later screen use, and between screen use and later depressive symptoms. However, a Random Intercept Cross Lagged Panel Model revealed no consistent support for a longitudinal association. The study highlights the importance of considering differential trajectories of depressive symptoms and specific forms of screen activity to understand these relationships.

## Introduction

In 2001, the World Health Organisation (WHO) predicted that childhood and adolescent mental health problems would become one of the leading causes of morbidity, mortality and disability worldwide by 2020. By 2012, ~20% of adolescents reported experiencing a mental health problem in any given year (WHO 2014), with depression being one of the most prevalent (Merikangas et al. 2009; see Patel 2017). Approximately 5–9% of adolescents are clinically depressed (see Goldfield et al. 2016) and one in four have experienced a depressive episode by the end of adolescence (see Hoare et al. 2016), with females twice as likely as males to experience depressive episodes (Hankin et al. 2007). With such high rates of depression among adolescents, more research is necessary to understand the trajectory of depressive symptoms and the effects of factors that might predict changes in symptomatology.

A meta-analysis of epidemiological studies (Costello et al. 2006) estimated the point prevalence rate of major depression among 13–18-years as 5.6%, while Bertha and Balázs (2013) reported 9–16% of 14–16 year olds experience sub-clinical levels of depressive symptoms (i.e., the presence of clinically relevant depressive symptoms that do not meet the full criteria of a major depressive episode). These sub-clinical levels increase the risk of depression and other psychopathology in adulthood considerably (Balázs et al. 2013; Bertha and Balázs 2013). These data are of major concern given that earlier onset of depression in youth is associated with drug and alcohol abuse, risky sexual behaviour and a poorer clinical course over the lifespan (see Werner-Seidler et al. 2017). Moreover, elevated rates of depressive symptoms among adolescents increases risk for comorbid health problems (see Vannucci and Ohannessian 2017) and is a central risk factor for suicide (Merikangas and Avenevoli 2002), which is the second leading cause of death in youth. However, while considerable variation in depressive symptom trajectories during adolescence exist (Garber and Cole 2010; Wickrama et al. 2008), the reciprocal relationships between these trajectories and other factors are not well understood.

### Trajectories of Depressive Symptoms

Ellis et al. (2017) reviewed 18 studies and found that multiple trajectories of depressive symptoms, primarily three (e.g., Chaiton et al. 2013; Diamantopoulou et al. 2011; Mezulis et al. 2014; Yaroslavsky et al. 2013) or four (e.g., Ellis et al. 2017; Reinke et al. 2012; Stoolmiller et al. 2005; Vannucci and Ohannessian 2017) provided best model fit. A common trajectory across all of these studies was a large group with stable and low levels of depressive symptoms, often accompanied by groups with moderate or high numbers of symptoms. However, there is a great deal of variation in the extent to which these latter two groups are characterised by increasing or decreasing symptomatology. The existence of different trajectory groups is likely to reflect the heterogeneity of the experience and expression of symptoms of depression during adolescence (Ellis et al. 2017). This variation may be attributable to the complex interactions between biological, psycho-social, and environmental causes that lead to depressive symptomatology (see e.g., Rice et al. 2003; Sonuga-Barke 2010). Clearly identifying and describing different trajectories, and reporting on which environmental factors are associated with each, will have important intervention and prevention implications.

Depressive symptoms are episodic and more common in adolescence than in childhood, raising the possibility there may be particular risks that young people experience as they get older (Gomez-Baya et al. 2017). The emergence of screen media as a predominant activity in the lives of adolescents is posited as one such risk. The research is unequivocal that adolescence is a period of high screen media use, and mobile devices now allow young people to be constantly connected to multiple activities, often simultaneously (Houghton et al. 2015; Sampasa-Kanyinga and Chaput 2016). This increased accessibility and time spent engaged with electronic screen media on a daily basis has raised health related concerns (see American Academy of Pediatrics AAP. 2016). While work examining the relationship between symptoms of depression across adolescence and screen use exists, it has been restricted to a single, overall depressive trajectory for all young people (Gunnell et al. 2016). Given the extensive research showing different trajectories of depression across adolescence, it is important to extend this work by examining screen use according to the different trajectory classes of depressive symptoms among adolescents.

### Depressive Symptoms and Electronic Screen Media Use

Research examining the dynamic interplay between screen media use and depressive symptoms is relatively scarce, especially longitudinally (Maras et al. 2015). Since depression is a significant cause of morbidity in adolescents, and screen media use is highly pervasive, any longitudinal relationship would be significant in terms of widespread health risk (Jelenchick et al. 2013; Twenge et al. 2018). Much of the evidence on this debate is based, either implicitly or explicitly, on a social displacement model which assumes that time spent on devices is inversely related (in a linear fashion) to well being because of the associated reduction in real life interactions (Kraut et al. 1998). However, empirical support for this theory is, at best, patchy (see Suchert et al. 2015, for a review). Thus, to date, the evidence does not unambiguously support the asserted linear associations between screen time and subsequent depression, and the possibility of non-linear effects has not been widely considered. Non-linear effects would be consistent with the digital Goldilocks Hypothesis (Przybylski and Weinstein 2017) which posits that moderate screen use can be positive and adaptive in societies where screen use is common and normative. Over-engagement with screens may still displace other adaptive activities, but under-engagement can also be problematic because it may remove opportunities for young people to achieve positive goals such as peer relationships (see Przybylski and Weinstein 2017).

Evidence for the digital Goldilocks Hypothesis is reported in cross-sectional work relating to the positive mental well being of adolescents (Przybylski and Weinstein 2017). In addition, three studies (Do et al. 2013; Durkin and Barber 2002; Kim 2012) have reported a curvilinear relationship between specific screen activities (i.e., internet use or video game playing) and depressive symptoms where moderate screen use was associated with the lowest rates of depressive symptoms. The same was reported by Liu et al (2016) in their review of screen time and its associations with depression. Evaluating the possibility that non-linear effects exist is therefore important and can help distinguish between social displacement and digital Goldilocks accounts of the potentially negative effects of screen time with respect to symptoms of depression.

In addition to the issues concerning the form of any relationship between screen time and (mal)adjustment, studies have tended to focus on a single or a small number of screens or screen activities (e.g., see Houghton et al. 2015; Selfhout et al. 2009). However, adolescents utilise a range of screen-based devices (e.g., tablets, smartphones, consoles, etc) to engage in many screen activities (e.g., TV viewing, gaming, social networking, internet) (Houghton et al. 2015; Rideout et al. 2010). In addition, the extent to which young people engage in these activities differs across the 13–18 year age range: e.g., texting and social networking appears to increase then stabilise, while TV and videogame use remain stable (Coyne et al. 2018). The likely costs and benefits to young people of these activities may also differ, and each may therefore have unique patterns of association with symptoms of depression. For example, passive screen use, such as watching TV, may be most likely to conform to the displacement model because it is less interactive, more frequently solitary, and so potentially amplifies symptoms in a linear fashion (George et al. 2018). In contrast, social media use is more likely to bring benefits at moderate levels of use because it offers opportunities to develop new friendships and to maintain existing ones; however, when online friendships displace those that also exist offline, social media use may become problematic (Nowland et al. 2018). These are relevant given the established relationships between friendships and depressive symptoms (e.g., Giletta et al. 2011). It is therefore important that investigations consider key activities that young people engage in when examining the links with trajectories of depressive symptoms.

## Current Study

The first aim of the current study was to use longitudinal data to identify trajectories of depressive symptoms in adolescents and to consider associations that might exist between trajectory classes and screen use time. Previous research has most often found three or four trajectory classes to best describe the data, but nothing is yet known about the degree to which different forms of screen use might be associated with depressive symptom trajectory membership. Hypothesis 1 is that there will be three or four latent trajectory classes. Of these, one will represent a large group (i.e., majority of the sample) with consistently low levels of depressive symptoms, and at least two further groups with high/increasing symptoms and decreasing symptoms, respectively. An exploratory aim of the study is to describe patterns of screen use for each of the depression trajectories.

The second aim of the study was to use longitudinal data to evaluate whether there are associations between screen use and subsequent depressive symptomatology or between depressive symptomatology and subsequent screen use. This second aim will also involve evaluation of the *nature* of any relationships (linear vs. non-linear) and whether these vary according to specific screen activity (gaming, social media use, TV/passive screen use, and using the web). Both displacement theory and the digital Goldilocks Hypothesis have been invoked to explain possible links between screen use and adjustment outcomes and these carry with them specific expectations concerning the nature of the associations (either linear or non-linear respectively). Therefore, Hypothesis 2 is that there will be positive, reciprocal longitudinal associations between screen use and depressive symptoms, but whether these are linear or non-linear may differ according to specific screen activity.

## Method

### Participants and settings

Three cohorts of participants were recruited at wave 1 from Grades 5 (10/11 years of age: 276 males, 247 females), 7 (12/13 years of age: 371 males, 298 females), and 9 (14/15 years of age: 289 males, 268 females). There were five further data collection sessions over three academic school years (2013–2015). As students joined existing participating classes, they too were invited to participate. To be included in the final analysis participants were required to have completed at least two surveys from waves 2, 4, and 6. Consequently, 1,749 adolescents (936 males and 813 females) in total participated. There were higher levels of non-participation at waves 1 and 3 because some potential participants were unable to complete online surveys due to technical issues, which restricted data collection. Table 1 shows the sample distribution.

**Table 1:** Sample distribution

	*N*	%
Sex		
Male	936	53
Female	813	47
School grade		
Grade 5	523	30
Grade 7	669	38
Grade 9	557	32
Geographical location		
Urban	1350	77
Rural	399	23
Wave		
1	1384	79
2	1533	88
3	1384	79
4	1687	96
5	1615	92
6	1571	89
Number of waves completed by participants		
1	0	0
2	20	1
3	205	12
4	160	9
5	302	17
6	1062	61

Initially, 30 schools representative of the socioeconomic demographics of Perth, Australia, were randomly selected. Of these 25 agreed to participate. This number of schools increased as the study progressed because some students moved schools and were tracked to their new school, while for many students the transition from primary to high school increased the number of participating schools; the final number of schools involved at wave 6 was 38. Of these, 13 were state government primary schools (4 in rural locations), 20 were state government high schools (4 in rural locations), 1 was a state government district high school (a rural location catering for grades Kindergarten to 10) and 4 were non-government schools (K-12). These schools were located across a range of socio-economic status (SES) areas as indexed by their Socio-Economic Index for Areas (SEIFA) (Australian Bureau of Statistics 2011). Five primary schools were in low SES areas, 3 in mid SES areas, and 5 were in high SES areas. Of the 20 high schools, there were three in low SES areas, eight in mid SES areas and nine in high SES areas. The District High School was in a medium SES area and of the four non-government K-12 schools, all were in high SES areas.

#### Screen Based Media Use

The Screen Based Media Use Scale (SBMUS) (see Houghton et al. 2015) was completed online. On first accessing the SBMUS, it is clarified what screens refer to and participants are requested to indicate which screen devices they have used over the preceding week: iPod Touch, iPad, Mobile Phone, Laptop, TV, Gaming consoles (e.g., Xbox), Portable gaming (e.g., Nintendo DS), and Computer. Using an interactive slide bar that measures screen use in hours and minutes, participants estimate the total amount of time they spend on screens (including use both inside and outside school) on a typical weekday from waking up until the time they go to bed. This is repeated for a typical weekend day. Examples of 20 different screen activities are then provided (e.g., Watched TV, Twitter, Shooter Game [e.g., Call of Duty], Online shopping) and participants indicate whether they have, or have not, engaged in each specific activity in the preceding week. Depending upon their responses to these items, the respondents then estimate the time they spent on a typical weekday and a typical weekend day (as used for total screen time) on gaming, social media use, TV/passive screen use, and web use. Each section has a definition and an illustrated image of what the section refers to.

Employing a sub-sample of 174 young people, Houghton et al (2015) assessed test-retest reliability of this measure across a six-month period. Overall reliability was good (*r* = .50, *N* = 174) and this did not differ by gender (*r*_boys_ = .51, *n* = 91; *r*_girls_ = .53, *n* = 82). Reliability varied somewhat across Grades 3 (*r* = .49, *n* = 33), 5 (*r* = .60, *n* = 44), and 7 (*r* = .52, *n* = 51). However, test-retest reliability was most problematic amongst the oldest group, those in Grade 9 (*r* = .19, *n* = 46). This was considered to be due to those young people taking examinations during that period.

To ensure a comprehensive coverage of screen use across the six administrations, surveys were administered at different times: wave 1, August/September 2013; wave 2, November/December 2013; wave 3, March/April 2014; wave 4, August/September 2014; wave 5, March/April 2015; and wave 6 August/September 2015. A large number of items included in waves 1 and 3 were for the development and validation of the SBMUS and the Adolescent Preoccupation with Screens Scale (Hunter et al. 2017). Therefore, to manage the burden on both individual participants and the school administrators, not all of the scales were administered in every wave.

Average screen use was a weighted average of weekday and weekend use, and both scores within each year (2013, 2014, 2015) were averaged. Weekday and weekend time use was weighted in the ratio 5:2. Where a young person participated in only one of the two waves used to calculate time use for that time period, the data for the wave the young person participated in was used.

#### Depressive Symptoms

The Children’s Depression Inventory 2 *(CDI 2)* (Kovacs 2004) is a brief self-report assessment of cognitive, affective and behavioural symptoms of depression in children and adolescents aged 7–17 years (Kovacs 2004). The CDI 2 consists of 12 items, each with three separate sentence response options which describe participants’ feelings and ideas over the past two weeks (e.g., I am sad once in a while, I am sad many times, I am sad all the time). To allow for appropriate age (7–12 years of age, 13–17 years of age) and sex (M/F) comparisons, total raw scores were converted to a standardised *T* score (mean of 50, SD = 10). The CDI 2 has demonstrated good reliability, and discriminant and convergent validity (Hodges 1990). Cronbach’s alpha has been reported as follows: for the overall total sample .82; the values among individual age and sex groupings ranging from .77 to .85; and test-retest reliability ranging from .76 to .92, indicating excellent temporal stability (see Kovacs 2004). The CDI 2 data were drawn from waves 2, 4, and 6. The wave 2 *T* score was used as the Year 1 (or baseline) measure (*α* = .80), wave 4 *T* score as the Year 2 measure (*α* = .84), and the wave 6 *T* scores were used as the Year 3 measure (*α* = .84).

### Procedure

The Human Research Ethics Committees of The University of Western Australia and the Western Australian Department of Education granted permission to conduct this research. Information sheets and consent forms were sent to parents of students explaining that involvement in the research consisted of multiple data gathering over approximately three school years. Informed consent was obtained from all individual participants included in the study. The SBMUS and CDI-2 were subsequently completed by participants via an online survey during regular school hours. All participants were provided with a unique identification code, which allowed them to log on to the survey at each administration. To ensure that the correct code was used it was given to each participant immediately prior to each administration. This unique code also ensured that all information provided was confidential and that data could be linked across waves via these codes for the purposes of data analysis.

School principals nominated one teacher to be responsible for liaising with the researchers and administering the survey at each time point. These teachers each received written instructions to ensure standardisation of administration procedures. The electronic survey remained open for approximately four weeks across each of the test administrations, which took into account each of the four academic school terms. The stringent monitoring of the survey administrations resulted in attrition rates being maintained below 6% per data collection period.

### Data Analysis

The analysis proceeded in three main steps. The first step involved identifying latent trajectories in depression symptomatology by fitting a latent growth curve model to the trajectories of the CDI 2 *T* scores. Once the latent trajectories were identified, the association between each of the trajectories and average screen time for each year was examined. The final stage involved fitting a Random Intercept Cross Lagged Panel Model (RI-CLPM: Hamaker et al. 2015) to examine the reciprocal longitudinal relationship between depressive symptoms and screen use.

The latent growth curve model was fitted using MPlus software, Version 7.4 (Muthén and Muthén 1998-2012). A latent growth curve model is a technique for examining individual differences in trajectories over time. It uses a latent variable structural equation-modelling framework in which there is a categorical latent variable that represents group membership, where each group has different shaped growth curves. The purpose is to identify individuals who have normative and non-normative developmental trajectories. Latent growth curve models allow for the dependence in measurements of the same person over time (Muthén and Muthén 2000). These models also adjusted for age and sex of the young person. The optimal number of latent groups can be tested using the Lo-Mendell-Rubin adjusted likelihood ratio test (Lo et al. 2001) and the bootstrapped parametric likelihood ratio test (McLachlan et al. 2003).

**Fig. 1:**
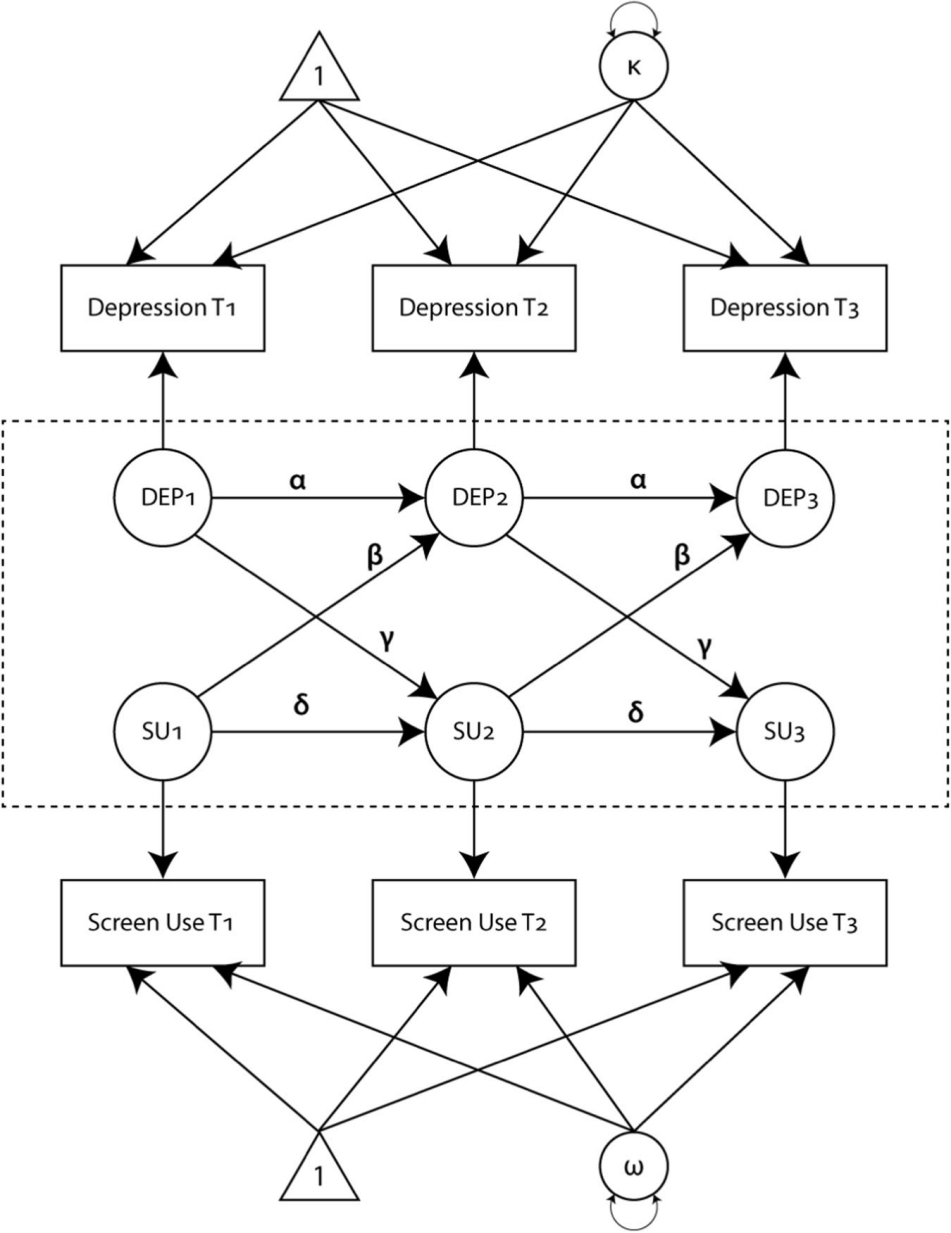
Random intercept cross-lagged panel model (RI-CLPM). Triangles represent constants (which define the mean structure), rectangles represent observed variables, and circles represent latent constructs. The terms κ and ω represent each individual’s trait-like deviations from the overall means. *α* and *δ* represent autoregressive parameters, and *β* and *γ* represent the cross-lagged regression parameters

To test whether changes in screen use are predictive of future changes in depression scores or vice-versa, the RI-CLPM of Hamaker et al (2015) was used. This extends the traditional Cross Lagged Panel Model (CLPM) by separately modelling the trait-like time-invariant components of each concept separately from the time varying component. Specifically, while some children are likely to use screens more than others, of specific interest is whether increasing screen use is associated with subsequent change in depressive symptoms or vice versa. This model is illustrated in Fig. 1. The primary purpose of this model is to examine whether symptoms of depression and screen use influence each other. This is illustrated in the central part of Fig. 1 in the dotted box. The cross-lagged regression parameter *β* measures the extent to which individual change in symptoms of depression is associated with the individual’s prior screen use. The cross-lagged regression parameter *γ* instead measures the extent to which change in screen use is associated with symptoms of depression in the previous time period. The substantive parameters of interest in this model are indicated by *α*, *β*, *γ*, and *δ* in Fig. 1. The autoregressive parameters *α* (the relationship between depression scores across time periods) and *δ* (the relationship between screen use across time periods) indicate the degree of stability in symptoms of depression or screen use over time. The cross-lagged regression parameters *β* (screen use at time 1 predicting symptoms of depression at time 2) and *γ* (symptoms of depression at time 1 predicting screen use at time 2), are the indicators of whether either variable is associated with changes in the other variable in the subsequent wave.

The model was fitted using MPlus software, Version 7.4 (Muthén and Muthén 1998-2012). For both symptoms of depression and screen use, within person changes over time were separated from stable between-person differences by including random intercepts. Separate models were fitted for total screen use and for each of the four main types of screen use activities measured in the study. While not a requirement of the RI-CLPM, the autoregressive and cross-lagged correlations were fixed to be the same between Year 1 and Year 2 as between Year 2 and Year 3. Because the primary RI-CLPM included children with a range of ages and developmental stages in wave 1, there was no a priori reason to believe there would be different effects between year 1 and year 2, and year 2 and year 3.

Recognising that screen use varies by age and sex, separate models were also fitted for each of the three age groups and both sexes. As the RI-CLPM uses a linear regression component to model the cross-lagged effects, we first examined the relationship between screen use and symptoms of depression to check for any evidence of a non-linear relationship. Initially, the distribution of depression scores within binned categories of screen use time using boxplots were examined. A generalised additive regression model was also fitted to the relationship between CDI 2 *T* score and screen use in the same time period. Generalised additive models fit a smooth function to the relationship without imposing any particular shape to the relationship. This was tested separately for both genders and each of the three grade cohorts.

As a final test of the possibility of a non-linear relationship between screen use and symptoms of depression, a threshold effect model (Hansen 1999) was fitted. Specifically, this model fits one linear parameter below some threshold value and another parameter above the threshold. For example, if screen use were not associated with developing symptoms of depression when screen use was low or in a typical range, but high screen use was associated with developing symptoms of depression, this model might be appropriate. Hansen (1999) proposes fitting this model by testing a large range of possible values for the threshold, and choosing the value that produces the best model fit.

## Results

### Trajectories in symptoms of depression over time

The latent growth curve model examining trajectories in depression scores (CDI 2 *T* scores) identified 3 latent groups as the optimal number of groups (Lo-Mendell-Rubin adjusted likelihood ratio test 3 vs 2 classes *p* < .001, 4 vs 3 classes *p* = 0.492; bootstrapped parametric likelihood ratio test 3 vs 2 classes *p* < .001, 4 vs 3 classes *p* = 1.000). In addition, using the Bayesian Information Criterion (BIC) and sample-size adjusted BIC the three group solution was a better fit than both the two group solution and the four group solution (adjusted BIC = 35135 for two group solution, 34684 for three group solution, 35081 for four group solution). Another consideration in model fit is the classification quality. The latent growth curve model estimates posterior probabilities for each individual belonging to each class. Entropy summarises classification quality. It ranges from 0 to 1 with values closer to 1 indicating clear classification. The entropy of the 3-group model was 0.87, and over 80% of the individuals in the study were assigned to a class with greater than 90% probability.

**Fig. 2:**
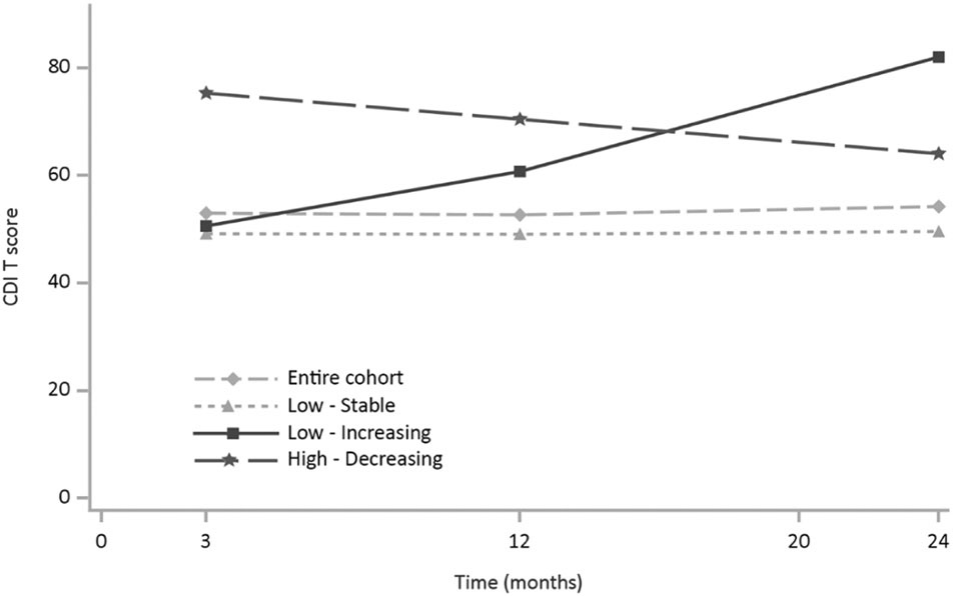
Depression (CDI 2 *T* score) by time and latent group

Average CDI 2 *T* scores in each of the three classes are shown in Fig. 2. The model identified one group of young people with CDI 2 *T* scores in the low range, suggesting no evident difficulties and little change over time. The average CDI 2 *T* score in this group was 49 in Year 1, 49 in Year 2 and 50 in Year 3. This group comprised 79.9% of the sample. This group was labelled “*Low- Stable*” and is indicative of young people who do not have significant symptoms of depression at any time period. A second group was identified who commenced with high CDI 2 *T* scores of 75 but showed improvement (i.e., reductions) over time (12.8% of the sample), and this group was labelled “*High- Decreasing*”. In this group, the mean CDI 2 *T* score in Year 2 was 70 and in Year 3 64. The third group had CDI 2 *T* scores around average at the first time point but developed worse symptoms of depression scores over time (7.3% of the sample). The mean CDI 2 *T* score in this group was 51 in Year 1, 61 in Year 2 and 82 in Year 3. This group was labelled “*Low - Increasing*”. The model did not constrain the trajectories to be linear, although the optimal solution appears close to linear across the three years of measurement. Where missing data occurred for individual waves, this was handled using maximum likelihood estimation under the assumption of data missing at random (Little and Rubin 2014).

### Association between trajectories of symptoms of depression and screen use

Substantial associations were observed between time spent using screens and depression symptom trajectories (see Table 2). For males with *Low - Stable* Depression the average Total screen time changed very little (from 3 h 1 min per day at Year 1 to 3 h 14 min per day in Year 3, an increase of 13 min per day (95% CI: 4 m, 23 m, *p* = .006)). This was also the case for females with *Low - Stable* Depression (from 4 h 13 min per day in Year 1 to 3 h 53 min per day in Year 3, a decrease of 21 min per day (95% CI: 7 m, 34 m, *p* = .003). In contrast, males who had *Low - Increasing* trajectories showed a substantial increase in average Total screen time, rising from 3 h 11 min to 4 h 58 min per day, an increase of 1 h 47 min (95% CI: 51 m, 2 h 43 min, *p* < .001), while for females it changed from 4 h 02 min to 5 h 11 min per day, an increase of 1 h and 09 min per day (95% CI: 0 h 13 m, 2 h 05 m, *p* = .016). For the *High – Decreasing* depression group, males average Total screen time started high (3 h 57 min per day) and remained that way throughout (4 h 19 min per day in Year 3), a difference of 21 min per day (95% CI: −14m, 56 m, *p* = .232); girls evidenced the same pattern, though with higher average Total screen time (starting at 4 h 43 min, rising to 5 h 3 min per day in Year 3, a difference of 20 min per day (95% CI: −6m, 46 m, *p* = .138)).

**Table 2:** Average time (in hours and minutes) spent on screen activities, by depression trajectory

Screen activity	Depression trajectory	Males			Females		
		Year 1	Year 2	Year 3	Year 1	Year 2	Year 3
Social media	Low - stable	0 h 50 m	0 h 57 m	1 h 10 m	1 h 33 m	1 h 34 m	1 h 59 m
	Low - increasing	1 h 29 m	1 h 47 m	2 h 58 m	2 h 14 m	2 h 09 m	2 h 50 m
	High - decreasing	1 h 31 m	2 h 02 m	2 h 12 m	2 h 50 m	2 h 51 m	3 h 25 m
Gaming	Low - stable	1 h 44 m	1 h 34 m	1 h 26 m	1 h 04 m	0 h 47 m	0 h 32 m
	Low - increasing	1 h 57 m	2 h 03 m	3 h 15 m	1 h 32 m	1 h 22 m	1 h 34 m
	High - decreasing	3 h 01 m	2 h 48 m	2 h 46 m	1 h 57 m	1 h 25 m	1 h 23 m
Web	Low - stable	1 h 13 m	1 h 17 m	1 h 22 m	1 h 54 m	1 h 47 m	1 h 58 m
	Low - increasing	1 h 32 m	2 h 02 m	2 h 55 m	2 h 05 m	2 h 13 m	2 h 41 m
	High - decreasing	1 h 53 m	2 h 04 m	2 h 12 m	2 h 30 m	2 h 24 m	2 h 28 m
TV/passive	Low - stable	2 h 01 m	1 h 51 m	1 h 50 m	2 h 39 m	2 h 30 m	2 h 26 m
	Low - increasing	2 h 28 m	2 h 33 m	4 h 01 m	3 h 06 m	3 h 07 m	3 h 40 m
	High - decreasing	2 h 42 m	2 h 38 m	2 h 50 m	3 h 34 m	3 h 18 m	3 h 32 m
Total screen time	Low - stable	3 h 01 m	3 h 03 m	3 h 14 m	4 h 13 m	3 h 50 m	3 h 53 m
	Low - increasing	3 h 11 m	3 h 36 m	4 h 58 m	4 h 02 m	4 h 26 m	5 h 11 m
	High - decreasing	3 h 57 m	4 h 33 m	4 h 19 m	4 h 43 m	4 h 56 m	5 h 03 m

With reference to average Total time spent on specific screen activities, males who had *Low - Increasing* depression symptom trajectories increased across all four types. Most time was spent in Year 3 (when their depression *T* score was highest) on TV/Passive screen use (an average of 4 h per day) though the remaining three activities were also relatively high at around 3 h per day. Both males and females who were *High - Decreasing* depression symptoms spent more time on gaming, web use, and TV/Passive screen use than both other depression symptom trajectory groups. By Year 3, it was the *Low – Increasing* depression symptom trajectory group who evidenced most screen use on these activities, although the *High - Decreasing* depression symptom trajectory group were still higher than the *Low – Stable* trajectory group. With regards to social media use, all participants reported spending more time on this activity each day as they progressed from Year 1 to Year 3. The most marked increase here was among the males in the *Low – Increasing* depression symptom trajectory group who doubled their time spent on social media (from 1 h 29 min to 2 h and 58 min, an increase of 1 h and 29 min per day (95% CI: 0 h 40 m, 2 h 17 m, *p* < .001). Supplementary Tables S.1-S.3 show the time spent on all screen activities and Total screen time, according to the three depression trajectories by sex and school grade.

### Relationship between symptoms of depression and screen use

**Fig. 3:**
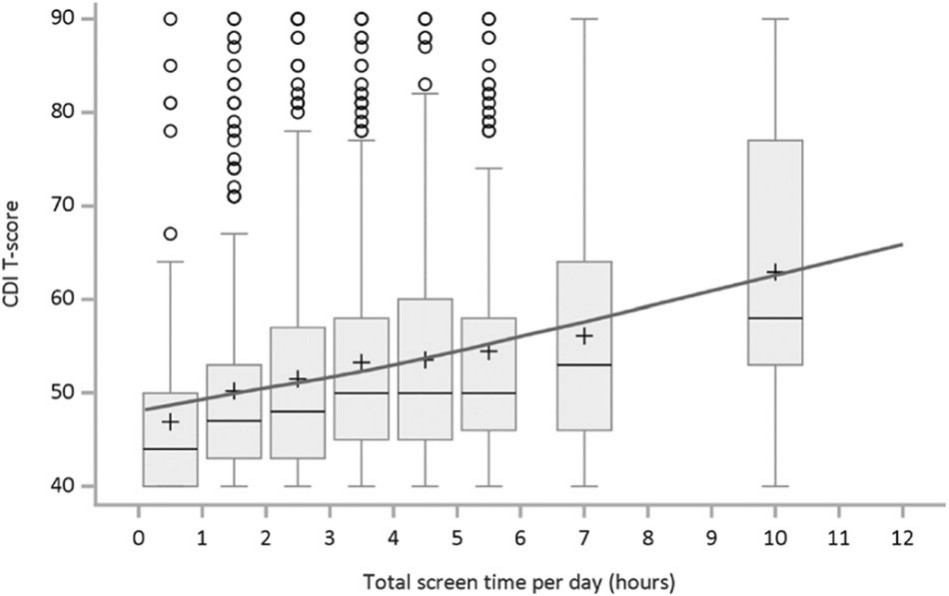
Distribution of depressive symptoms (CDI *T* score) and predicted mean depression score by level of screen use. Note: Regression line computed using generalised additive models, allowing for possible non-linear relationship. Non-linear component was non-significant (*p* = .981)

Before testing for reciprocal effects using the RI-CLPM, we tested for non-linearity in the relationship between depression and screen use. Figure 3 shows the distribution of CDI 2 *T* scores within binned categories of average Total hours of screen time, and a non-linear regression using a generalised additive model. This found no evidence of any non-linearity in the relationship, and the non-linear component of the regression was non-significant (*p* = .981). Similar patterns were observed for each grade cohort and both sexes, suggesting that in these data it is appropriate to model a linear relationship between symptoms of depression and screen use.

The cross-correlations between symptoms of depression and screen use were modelled using the RI-CLPM. Standardised model results are shown in Table 3 for the relationships between Total screen use and symptoms of depression and between each of the four screen activities and symptoms of depression.

**Table 3:** Cross-lagged standardised effects (RI-CLPM) between depression and time spent using screens

	*b*	SE	*p*	Model fit
Total screen time				
Depression → Depression (*α*)	**0.231**	**0.061**	**.000**	RMSEA = 0.036
Depression → Screen use (*γ*)	**0.147**	**0.049**	**.003**	*χ*^2^ = 19.3 *p* = .007
Screen use → Depression (*β*)	**0.128**	**0.057**	**.024**	ICC_Depression_ = 0.31
Screen use → Screen use (*δ*)	**0.375**	**0.067**	**.000**	ICC_Screen Time = _0.53
Social media				
Depression → Depression (*α*)	**0.217**	**0.060**	**.000**	RMSEA = 0.010
Depression → Screen use (*γ*)	0.036	0.050	.474	*χ*^2^ = 7.9 *p* = .340
Screen use → Depression (*β*)	0.008	0.051	.873	ICC_Depression_ = 0.34
Screen use → Screen use (*δ*)	**0.208**	**0.060**	**.001**	ICC_Social media = _0.48
Gaming				
Depression → Depression (*α*)	**0.210**	**0.060**	**.001**	RMSEA = 0.050
Depression → Screen use (*γ*)	0.033	0.045	.459	*χ*^2^ = 31.3 *p* = .001
Screen use → Depression (*β*)	0.041	0.050	.416	ICC_Depression_ = 0.33
Screen use → Screen use (*δ*)	**0.318**	**0.056**	**.000**	ICC_Gaming = _0.40
TV/passive				
Depression → Depression (*α*)	**0.210**	**0.061**	**.001**	RMSEA = 0.029
Depression → Screen use (*γ*)	0.042	0.045	.356	*χ*^2^ = 17.1 *p* = .029
Screen use → Depression (*β*)	0.054	0.045	.230	ICC_Depression_ = 0.33
Screen use → Screen use (*δ*)	**0.192**	**0.050**	**.000**	ICC_TV = _0.37
Web				
Depression → Depression (*α*)	**0.218**	**0.059**	**.000**	RMSEA = 0.032
Depression → Screen use (*γ*)	0.038	0.046	.410	*χ*^2^ = 19.3 *p* = .013
Screen use → Depression (*β*)	**0.090**	**0.044**	**.042**	ICC_Depression_ = 0.33
Screen use → Screen use (*δ*)	**0.172**	**0.052**	**.001**	ICC_Web = _0.53

NB, rows in bold reflect significant parameter estimates

### Cross-lagged effects (RI-CLPM) between symptoms of depression and time spent using screens

For Total screen use, significant cross-lagged effects were found. In the standardised results, there was a statistically significant association between symptoms of depression in one wave and screen use in the subsequent wave and also between screen use at one wave and symptoms of depression in the subsequent wave (Table 3 shows the standardised regression results). A one hour increase in screen time was associated with a 0.76 point increase in the depression *T* score in the subsequent wave (unstandardised *β* = 0.76, SE = 0.33, *p* = .021). Reciprocally, for each one point increase in depression *T* score, total screen time in the subsequent wave increased by 0.024 h (i.e., just under 2 min) (unstandardised *γ* = 0.024, SE = 0.01, *p* = .002). Assuming linear effects, this suggests that an increase in screen time of approximately 13 h would be required to evidence a young person moving from average (*T* score = 50) into the clinical range (*T* score = 60) for depression. Thus, while significant, these effects are small. With reference to individual screen use activities, only one of the eight cross-lagged effects was statistically different from zero, with increase in time spent on the Web in one wave associated with an increase in symptoms of depression in the following wave.

The intra class correlation coefficients for depression averaged about 0.33 and for screen time ranged between 0.37 and 0.53 across the different types of screen use. These relatively high values confirm the persistence of depression and screen use patterns from one time to the next. A separate RI-CLPM was run for males and females (Table S.4). No significant cross-lagged effects were found for females. For males significant, reciprocal cross-lagged effects were found for depressive symptoms and both total screen time and for time spent using social media. There was also a significant positive association between boys’, but not girls’, web use and their later depressive symptoms. Separate models were fitted for each of the four types of screen activity for each Grade in school by sex and the results are shown in supplementary Tables S.5–S.7. Only one cross-lagged effect was found to be significantly different from zero, and this was for depression as a predictor of social media screen use in the next wave for Grade 9 males. No adjustments were made for multiple testing. However, given the large number of statistical tests included in these models, and the comparatively low number of non-zero coefficients found, it is reasonable to conclude that the one significant finding evident in these models could be due to chance. This is consistent with the findings of the changes in screen use over time for the three depression symptom trajectory groups, thereby suggesting that for children whose level of depression increases over time, screen use increases commensurately at the same time, neither leading nor lagging.

To further investigate the possibility of a non-linear association between screen use and symptoms of depression, we fitted separate threshold panel models for each screen use type. No evidence was found of there being different effects below or above a threshold for any of these models. There was insufficient power to fit threshold models by grade and sex. Two sensitivity analyses were conducted. At first we refitted the RI-CLPM using reported screen time at Waves 2, 4 and 6 only instead of averaging the values from Waves 1 and 2, 3 and 4, 5 and 6, respectively (see S.8). Results were similar to the Main model (Table 3) with significant cross-lagged effects for total screen time, however unlike in the main model in this model no significant cross-lagged effect was found for time spent on the web. As a final sensitivity analysis, we refit the main RI-CLPM without constraining the effects between Year 1 and Year 2 and between Year 2 and Year 3 to be equal (see Table S.9). Only modest differences within the bounds of random variation were observed between the two time periods.

In this study, all the data exclusions and manipulations relevant to the hypotheses addressed in this paper are reported. These data were drawn from a larger study that examined the extent of screen media use and its problematic nature among adolescents.

## Discussion

Depression is one of the most prevalent mental health problems that adolescents experience (Merikangas et al. 2009; see Patel 2017), and even sub-clinical levels increase the risk of later psychopathologies (Balázs et al. 2013; Bertha and Balázs 2013). Screen media use is almost ubiquitous during adolescence and may act as a risk for the development of raised symptomatology. Assessing young people aged 10–17 years, and considering multiple screen based activities, this study identified three different trajectories of adolescent depressive symptomatology and the characteristic patterns of screen use for each. In addition, the degree to which there were cross-lagged associations between screen use and depressive symptomatology was assessed. There was no evidence to support the digital Goldilocks Hypothesis (Przybylski and Weinstein 2017) which proposes there is a “just right” level of screen use that is adaptive. There was some limited evidence supporting a social displacement model (Kraut et al. 1998), though even here our conclusion is that any relationships between screen use and depressive symptomatology appear to be, at best, small.

The present research extends the work of what appears to be the only existing study to examine screen use and the trajectory of depressive symptoms in adolescence (Gunnell et al. 2016). However, that study only considered a single trajectory of depressive symptoms whereas our study investigated multiple trajectories of depressive symptoms (reflecting previous work on trajectories of depressive symptomatology). We also assessed screen use across a range of screen-based devices (including mobile devices, e.g., smartphones and tablets) and screen activities (gaming, social networking, web use, passive screen use). In addition, we extended the understanding of links between these variables by both evaluating the differences in screen activities for young people in different trajectory classes and looking at the shape and degree of prospective reciprocal relationships between screen activities and depressive symptomatology. In doing so, the present study provides a more comprehensive picture of the ways in which screen use and depressive symptoms are associated during adolescence.

Supporting Hypothesis 1, that there would be either three or four trajectory classes of depressive symptoms, the present study identified three classes: *Low - Stable*, *High - Decreasing*, and *Low - Increasing*. This reflects previous work, which also identified three groups (e.g., Chaiton et al. 2013; Diamantopoulou et al. 2011; Mezulis et al. 2014; Yaroslavsky et al. 2013). Consistent with all of the studies that identified three (or four) depressive symptom trajectories, the present work also found one large subgroup of adolescents (79.9%) that displayed consistently low levels of depressive symptoms over time. Most other studies to date identified at least two other subgroups that were characterised by trajectories with increasing levels of depressive symptoms over time, or symptoms that declined over time. In the present study, these latter two groups comprised almost 21% of adolescents whose trajectories of depressive symptoms either escalated relatively quickly to severe levels (CDI 2 *T* scores rising from 50 to > 80) or started high and, although decreasing, remained in the problematic range of symptoms (all CDI 2 *T* scores > 60). This reflects research showing that up to 20% of young people report depression or sub-clinical levels of depressive symptoms (Bertha and Balázs 2013; Costello et al. 2006).

Substantial associations were apparent in the trajectories of depressive symptoms and patterns of time spent on screen use. For young people in the *Low - Increasing* depression trajectory there were notable increases in time spent on screen use over a two-year period (boys increased by 1 h 47 min and girls increased by 1 h and 9 min). In contrast, those young people with *Low – Stable* depressive symptoms reported stable time spent on screen use (boys increased by 13 min and girls *decreased* by 20 min) across the same period. Finally, for the *High – Decreasing* depression trajectory group, time spent on screen use was also relatively stable (boys increased by 22 min and girls increased by 20 min). These first two trajectory groups suggest that screen use and symptoms of depression are closely linked, but the final trajectory group (*High – Decreasing* depression) implies this may not be the case. Specifically, while initially high levels of depressive symptoms reduced, this was not accompanied by similar reductions in screen time, though it is important to note that, despite these reductions in depressive symptoms, gross levels of symptomatology remained in the problematic range (all CDI 2 *T* scores > 60) for this trajectory throughout. These results argue against a straightforward positive relationship between depressive symptoms and screen media use as documented in previous research (e.g., Maras et al. 2015; Suchert et al. 2015).

When examining specific screen activities reported by the three depressive symptom trajectory groups, the *Low - Increasing* depression trajectory again evidenced important sex differences. Among males, the time spent each day on three of the four activities almost doubled from Year 1 to Year 3 of the study. Social media use increased from 1 h 29 min to 2 h 58 min per day, Passive screen use increased from 2 h 28 min to 4 h 1 min per day, and Web use from 1 h 32 min to 2 h 55 min. Although not double, Gaming also increased from 1 h 57 min to 3 h 15 min per day. Females, in contrast, only increased by between 2 to 36 min across the four activities. Such large, relatively quick increases in the amount of time being spent on screens by adolescent males may potentially alert family members that depressive symptoms could also be increasing.

Hypothesis 2 was that there would be positive, reciprocal longitudinal associations between screen use and depressive symptoms, and that whether these were linear or non-linear may differ according to specific screen activity. The possibility of screen use and symptoms of depression demonstrating a reciprocal relationship was directly evaluated using the Random Intercept Cross Lagged Panel Model (RI-CLPM) of Hamaker et al (2015). Unlike the traditional Cross Lagged Panel Design (which assumes there are no trait like individual differences that endure over time), the RI-CLPM attempts to disentangle the within-person process from stable between person differences that are likely to be present in the data. The findings revealed statistically significant, but small, cross-lagged effects for total screen time and symptoms of depression. This suggests the causal association between screen use and depression is very modest. This assertion is supported by the unstandardised results, which suggest that each one hour increase in screen time is only associated with a 0.76 point increase in the CDI 2 depression *T* score in the subsequent wave. Reciprocally, a 10-point increase in CDI 2 depression *T* score was associated with only a 20-min increase in total average screen time in the subsequent wave.

When the screen use activities were examined, only one of the eight cross-lagged effects was statistically different from zero, with an increase in time spent on the Web in one wave being predictive of an increase in depression in the following wave. As no adjustments were made for multiple statistical testing it is quite possible that a single significant result could be due to chance alone. It is also possible that important moderating variables were missed in our analyses. For example, there was no reciprocal associations between social media use and depressive symptoms when looking at the group as a whole, yet small positive effects were present for boys (but not girls) when this was assessed by sex. Future research may yet identify important moderating variables. For example, there is growing evidence regarding the impact of screen use on sleep, particularly sleep debt and disrupted sleep and its association with depression (Touitou et al. 2016; Woods and Scott 2016).

Contrary to the present findings, recent research reported clear links between screen media activities and higher levels of depressive symptoms among adolescents, with daily use of social media sites increasing levels of depressive symptoms by 13% (Twenge et al. 2018). However, Twenge et al report correlations between increases in screen use and increases in depressive symptoms over the period 2011–2016. They used data from a series of national surveys and did not have access to longitudinal data to examine whether individual changes in screen use were associated with depressive symptoms.

Gunnell et al. (2016) investigated the bidirectional relationship between screen time and health outcomes and found that symptoms of depression are independently related to screen time (and physical activity) both cross-sectionally *and* longitudinally. Although only one bidirectional finding related to physical activity and depression was evident, the results were promising because they indicated that levels of screen time at age 13.5 years are not indicative of changes in depressive symptoms and screen use time over time. The present study used the RI-CLP model and found only a very modest causal association, suggesting that most of the parallel increase in screen time observed among the *Low - Increasing* depression symptom trajectory was an association rather than a causal pathway. It therefore seems reasonable to conclude that the results of these models do not provide evidence of a causal link between screen use and subsequent changes in symptoms of depression, or vice versa. This is consistent with the findings of the changes in screen use over time for the three depression symptom trajectory groups, which suggested that for adolescents whose symptoms of depression increase over time (*Low - Increasing Depression*), screen use increases commensurately at the same time, neither leading nor lagging.

As our results found no longitudinal association, they do not support a causal link between screen use and depressive symptoms. There was however, a temporal association between depressive symptoms and screen use in those young people who developed depression over the course of the study. As such, significant increases in screen use time may indicate that a young person’s mental health is deteriorating. While not directly assessed in the current study, it is possible that some young people use screens in unproductive and unhelpful ways to cope with stressors encountered during this developmental period. Such increases in screen use could be a useful way of opening up conversations between adults and young people about mental health and about healthy and productive ways to cope with the demands placed upon today’s adolescents.

Our results offered no support for the Digital Goldilocks Hypothesis, which proposes that both low and high levels of screen use may be associated with poorer outcomes (Przybylski and Weinstein 2017). The small effects observed showing associations between increases in screen time and depressive symptoms does offer some limited support for a displacement model. This may indicate that a displacement model is appropriate when considering screen use and symptoms of depression. However, positive well being was assessed as an outcome by Przybylski and Weinstein (2017) and future research should therefore seek to directly assess whether the Digital Goldilocks Hypothesis is limited to positive outcomes.

It is acknowledged that the current study utilised a sample of adolescents from Western Australia and therefore results may not generalise to other populations. Furthermore, screen use, screen activities and depressive symptoms were all assessed using self-report. While subject to the usual limitation claims surrounding self-report, it is an effective means of obtaining an accurate insight into the subjective dispositions (depressive symptoms) that can be difficult to obtain from third parties such as teachers and parents (see Frick et al. 2009; Houghton et al. 2013). Furthermore, only young people themselves can report on screen use across an entire waking day given that this will include the use of many different screens in a range of contexts (home, school, friends’ houses, etc.) at different times.

The wider social context surrounding adolescents screen use, including their motives for using different types of screen activities was not considered in the present study. This is an important aspect of screen use not reflected in the overall measures of screen time and separate screen activities such as used here. Some popular games, for example, provide a context for adolescents in which to socialise, feel connected to friends and be creative (see Przybylski and Weinstein 2017), while others can motivate young people to engage in physical activity (O’Hara 2008). The reasons why young people engage with specific screen activities potentially influences the amount of time they spend on that screen activity, which in turn may affect their health status.

Nevertheless, the present study also has important strengths such as recruiting a large demographically representative sample, a six-wave longitudinal design with low levels of attrition, and the use of state-of-the-art techniques to identify trajectories and to test for reciprocal predictive effects. These advantages combine to present a convincing case for the lack of consistent association between depressive symptomatology and screen use among young people.

## Conclusion

Virtually all adolescents in developed countries access screen media on a daily basis, and increasingly through mobile devices either at home, school or at their friends’ homes. Up to 20% of adolescents also experience a mental health problem in any given year, with depression being one of the most debilitating. While prior studies have found associations between screen use and depression, the issue of a causal relationship between the two has received little attention. The one longitudinal study to examine bi-directionality (Gunnell et al. 2016) found initial levels of screen use and depression were not predictors of change in each other. However only a single depressive trajectory was produced for all young people and screen use was restricted to TV viewing, video game playing and computer use. Here, this work was extended by identifying multiple trajectories of depressive symptoms (Low - Stable, High - Decreasing, and Low – Increasing), and by identifying associations between these trajectories and patterns of screen use. Additionally, sex differences in specific screen use activities and trajectories of depressive symptoms were identified. Contrary to previous research (e.g., Maras et al. 2015; Suchert et al. 2015), the current research argues against a straightforward positive relationship between depressive symptoms and screen use.

No substantial evidence for a longitudinal association between screen use and depressive symptoms was identified, undermining the likelihood that there is a causal link between screen use and subsequent changes in depression, or vice versa. There was however, a temporal association found in those who experienced increases in depressive symptoms over the course of the study. This is a valuable contribution to existing research in that significant increases in screen use time may indicate that a young person’s mental health is deteriorating. While not directly assessed in the current study, some adolescents may use screens in unproductive and unhelpful ways to cope with the stressors they encounter. Such increases in screen use could be a useful way of opening up conversations between adults and young people about mental health and about healthy and productive ways to cope with the demands placed upon today’s adolescents. It might prove to be a useful marker for clinicians, educators and families in their concerns over their children and adolescents developing symptoms of adverse mental health.

## Electronic supplementary material

Supplementary Information
