# Supplementary material for: Reciprocal Relationships between Trajectories of Depressive Symptoms and Screen Media Use during Adolescence
Source: J Youth Adolesc. 2018 Jul 25;47(11):2453–67. doi: 10.1007/s10964-018-0901-y (PMC6208639; doi:10.1007/s10964-018-0901-y)
Supplement: Supplementary file 1 — Supplementary Information [file 10964_2018_901_MOESM1_ESM.docx]

Table S.1. Average time (in hours and minutes) spent on screen activities, by depression trajectory, for students in Grade 5 at Baseline

|  | | | | | | | | |
| --- | --- | --- | --- | --- | --- | --- | --- | --- |
| Screen activity | | Depression Trajectory | Males | | | Females | | |
|  |  |  | Year 1 | Year 2 | Year 3 | Year 1 | Year 2 | Year 3 |
| Social media | Low - Stable | | 0h 40m | 0h 34m | 0h 53m | 0h 43m | 0h 46m | 1h 15m |
|  | Low - Increasing | | 1h 02m | 0h 49m | 0h 55m | 0h 41m | 1h 04m | 1h 53m |
|  | High - Decreasing | | 0h 37m | 0h 54m | 1h 08m | 2h 08m | 1h 59m | 2h 49m |
| Gaming | Low - Stable | | 1h 59m | 1h 45m | 1h 43m | 1h 26m | 0h 59m | 0h 35m |
|  | Low - Increasing | | 2h 18m | 2h 51m | 1h 40m | 1h 39m | 1h 04m | 1h 48m |
|  | High - Decreasing | | 2h 36m | 1h 56m | 2h 15m | 3h 11m | 2h 14m | 2h 00m |
| Web | Low - Stable | | 1h 11m | 1h 01m | 1h 15m | 1h 18m | 1h 06m | 1h 26m |
|  | Low - Increasing | | 0h 59m | 0h 55m | 0h 47m | 0h 56m | 0h 57m | 2h 30m |
|  | High - Decreasing | | 1h 29m | 1h 10m | 1h 20m | 2h 23m | 1h 37m | 1h 40m |
| TV/Passive | Low - Stable | | 2h 03m | 1h 48m | 1h 44m | 2h 09m | 2h 02m | 1h 56m |
|  | Low - Increasing | | 2h 25m | 2h 33m | 2h 34m | 1h 33m | 2h 03m | 2h 08m |
|  | High - Decreasing | | 2h 12m | 1h 47m | 1h 54m | 3h 55m | 3h 01m | 3h 13m |
| Total screen time | Low - Stable | | 2h 37m | 2h 20m | 2h 57m | 2h 37m | 2h 20m | 2h 36m |
|  | Low - Increasing | | 2h 46m | 2h 57m | 2h 54m | 2h 01m | 2h 52m | 4h 23m |
|  | High - Decreasing | | 3h 04m | 2h 38m | 2h 46m | 3h 22m | 3h 54m | 3h 53m |

Table S.2. Average time (in hours and minutes) spent on screen activities, by depression trajectory, for students in Grade 7 at Baseline

|  | | | | | | | | |
| --- | --- | --- | --- | --- | --- | --- | --- | --- |
| Screen activity | | Depression Trajectory | Males | | | Females | | |
|  |  |  | Year 1 | Year 2 | Year 3 | Year 1 | Year 2 | Year 3 |
| Social media | Low - Stable | | 0h 43m | 0h 51m | 1h 01m | 1h 41m | 1h 48m | 2h 11m |
|  | Low - Increasing | | 1h 00m | 1h 06m | 3h 36m | 2h 29m | 2h 05m | 3h 28m |
|  | High - Decreasing | | 2h 13m | 2h 19m | 2h 13m | 2h 45m | 3h 10m | 3h 41m |
| Gaming | Low - Stable | | 1h 38m | 1h 29m | 1h 23m | 1h 00m | 0h 46m | 0h 26m |
|  | Low - Increasing | | 2h 26m | 1h 39m | 5h 24m | 1h 47m | 1h 51m | 1h 34m |
|  | High - Decreasing | | 3h 11m | 3h 13m | 2h 56m | 1h 13m | 0h 45m | 0h 46m |
| Web | Low - Stable | | 1h 07m | 1h 10m | 1h 12m | 1h 53m | 1h 51m | 1h 57m |
|  | Low - Increasing | | 1h 43m | 2h 48m | 4h 04m | 2h 41m | 2h 33m | 3h 00m |
|  | High - Decreasing | | 2h 05m | 2h 29m | 2h 16m | 2h 22m | 2h 25m | 2h 21m |
| TV/Passive | Low - Stable | | 1h 58m | 1h 45m | 1h 51m | 2h 47m | 2h 27m | 2h 28m |
|  | Low - Increasing | | 2h 42m | 2h 10m | 4h 45m | 3h 38m | 3h 32m | 4h 55m |
|  | High - Decreasing | | 2h 47m | 3h 02m | 3h 07m | 3h 12m | 3h 19m | 3h 20m |
| Total screen time | Low - Stable | | 2h 54m | 2h 56m | 3h 10m | 4h 12m | 4h 06m | 4h 07m |
|  | Low - Increasing | | 2h 53m | 2h 55m | 6h 19m | 4h 58m | 4h 40m | 6h 07m |
|  | High - Decreasing | | 3h 50m | 4h 52m | 4h 24m | 4h 47m | 5h 11m | 5h 15m |

Table S.3. Average time (in hours and minutes) spent on screen activities, by depression trajectory, for students in Grade 9 at Baseline

|  | | | | | | | | |
| --- | --- | --- | --- | --- | --- | --- | --- | --- |
| Screen activity | | Depression Trajectory | Males | | | Females | | |
|  |  |  | Year 1 | Year 2 | Year 3 | Year 1 | Year 2 | Year 3 |
| Social media | Low - Stable | | 1h 09m | 1h 28m | 1h 39m | 2h 17m | 2h 08m | 2h 29m |
|  | Low - Increasing | | 2h 02m | 2h 45m | 3h 41m | 3h 32m | 3h 35m | 2h 33m |
|  | High - Decreasing | | 1h 23m | 2h 32m | 2h 57m | 3h 27m | 3h 15m | 3h 39m |
| Gaming | Low - Stable | | 1h 37m | 1h 31m | 1h 14m | 0h 46m | 0h 35m | 0h 35m |
|  | Low - Increasing | | 1h 25m | 1h 55m | 2h 39m | 0h 53m | 0h 40m | 1h 18m |
|  | High - Decreasing | | 3h 07m | 2h 58m | 2h 56m | 1h 36m | 1h 19m | 1h 26m |
| Web | Low - Stable | | 1h 24m | 1h 41m | 1h 43m | 2h 31m | 2h 21m | 2h 30m |
|  | Low - Increasing | | 1h 43m | 2h 09m | 3h 16m | 2h 21m | 3h 02m | 2h 13m |
|  | High - Decreasing | | 1h 55m | 2h 14m | 2h 45m | 2h 42m | 2h 57m | 3h 10m |
| TV/Passive | Low - Stable | | 2h 02m | 2h 03m | 1h 56m | 3h 00m | 3h 02m | 2h 55m |
|  | Low - Increasing | | 2h 22m | 2h 47m | 4h 18m | 3h 47m | 3h 27m | 2h 46m |
|  | High - Decreasing | | 2h 56m | 2h 48m | 3h 09m | 3h 37m | 3h 30m | 4h 00m |
| Total screen time | Low - Stable | | 3h 33m | 3h 51m | 3h 42m | 5h 41m | 4h 53m | 5h 03m |
|  | Low - Increasing | | 3h 38m | 4h 21m | 5h 04m | 4h 34m | 5h 16m | 3h 46m |
|  | High - Decreasing | | 4h 35m | 5h 14m | 5h 17m | 5h 38m | 5h 38m | 5h 44m |

Table S.4. Cross-lagged standardized effects (RI-CLPM) between depression and time spent using screens, by gender

|  | Males | | | Females | | |
| --- | --- | --- | --- | --- | --- | --- |
|  | b | SE | *p* | b | SE | *p* |
| Total Screen Time | | | | | | |
| Depression 🡪 Depression (α) | **0.282** | **0.064** | **.000** | **0.337** | **0.064** | **.000** |
| Depression 🡪 Screen use (γ) | **0.187** | **0.046** | **.000** | 0.074 | 0.059 | .212 |
| Screen use 🡪 Depression (β) | **0.124** | **0.054** | **.021** | 0.092 | 0.060 | .126 |
| Screen use 🡪 Screen use (δ) | **0.392** | **0.045** | **.000** | **0.324** | **0.062** | **.000** |
| Social Media | | | | | | |
| Depression 🡪 Depression (α) | **0.301** | **0.059** | **.000** | **0.361** | **0.069** | **.000** |
| Depression 🡪 Screen use (γ) | **0.163** | **0.052** | **.002** | -0.061 | 0.052 | .238 |
| Screen use 🡪 Depression (β) | **0.150** | **0.047** | **.002** | -0.083 | 0.051 | .104 |
| Screen use 🡪 Screen use (δ) | **0.372** | **0.044** | **.000** | **0.284** | **0.061** | **.000** |
| Gaming | | | | | | |
| Depression 🡪 Depression (α) | **0.314** | **0.066** | **.000** | **0.325** | **0.079** | **.000** |
| Depression 🡪 Screen use (γ) | 0.045 | 0.047 | .338 | 0.038 | 0.045 | .404 |
| Screen use 🡪 Depression (β) | 0.040 | 0.053 | .451 | 0.050 | 0.048 | .302 |
| Screen use 🡪 Screen use (δ) | **0.351** | **0.046** | **.000** | **0.250** | **0.046** | **.000** |
| TV/Passive | | | | | | |
| Depression 🡪 Depression (α) | **0.311** | **0.065** | **.000** | **0.308** | **0.067** | **.000** |
| Depression 🡪 Screen use (γ) | 0.108 | 0.057 | .058 | 0.033 | 0.057 | .563 |
| Screen use 🡪 Depression (β) | 0.057 | 0.054 | .290 | 0.072 | 0.058 | .212 |
| Screen use 🡪 Screen use (δ) | **0.324** | **0.066** | **.000** | **0.232** | **0.056** | **.000** |
| Web | | | | | | |
| Depression 🡪 Depression (α) | **0.299** | **0.063** | **.000** | **0.324** | **0.066** | **.000** |
| Depression 🡪 Screen use (γ) | 0.058 | 0.050 | .246 | -0.022 | 0.059 | .707 |
| Screen use 🡪 Depression (β) | **0.115** | **0.053** | **.028** | 0.070 | 0.056 | .211 |
| Screen use 🡪 Screen use (δ) | **0.193** | **0.074** | **.009** | **0.159** | **0.057** | **.005** |

NB. rows in bold reflect significant parameter estimates.

Table S.5. Cross-lagged standardized effects (RI-CLPM) between depression and time spent using screens by gender for students in Grade 5 at baseline

|  | Males | | | Females | | |
| --- | --- | --- | --- | --- | --- | --- |
|  | b | SE | *p* | b | SE | *p* |
| Total Screen Time | | | | | | |
| Depression 🡪 Depression (α) | -0.108 | 0.235 | .646 | **0.325** | **0.137** | **.018** |
| Depression 🡪 Screen use (γ) | 0.032 | 0.103 | .757 | -0.054 | 0.092 | .555 |
| Screen use 🡪 Depression (β) | 0.361 | 0.308 | .241 | 0.002 | 0.116 | .986 |
| Screen use 🡪 Screen use (δ) | **0.748** | **0.164** | **.000** | **0.541** | **0.198** | **.006** |
| Social Media | | | | | | |
| Depression 🡪 Depression (α) | -0.053 | 0.227 | .816 | **0.325** | **0.135** | **.016** |
| Depression 🡪 Screen use (γ) | -0.219 | 0.184 | .233 | -0.222 | 0.213 | .296 |
| Screen use 🡪 Depression (β) | 0.013 | 0.149 | .929 | -0.106 | 0.121 | .381 |
| Screen use 🡪 Screen use (δ) | 0.047 | 0.171 | .783 | -0.151 | 0.246 | .539 |
| Gaming | | | | | | |
| Depression 🡪 Depression (α) | 0.149 | 0.190 | .433 | 0.183 | 0.094 | .051 |
| Depression 🡪 Screen use (γ) | -0.043 | 0.113 | .700 | 0.007 | 0.094 | .937 |
| Screen use 🡪 Depression (β) | -0.036 | 0.140 | .799 | -0.007 | 0.097 | .944 |
| Screen use 🡪 Screen use (δ) | **0.300** | **0.126** | **.017** | **0.467** | **0.122** | **.000** |
| TV/Passive | | | | | | |
| Depression 🡪 Depression (α) | 0.163 | 0.196 | .405 | 0.187 | 0.099 | .060 |
| Depression 🡪 Screen use (γ) | 0.054 | 0.130 | .678 | -0.039 | 0.111 | .729 |
| Screen use 🡪 Depression (β) | 0.000 | 0.140 | .999 | 0.056 | 0.089 | .528 |
| Screen use 🡪 Screen use (δ) | 0.150 | 0.124 | .227 | 0.051 | 0.143 | .722 |
| Web | | | | | | |
| Depression 🡪 Depression (α) | 0.137 | 0.187 | .464 | 0.187 | 0.098 | .056 |
| Depression 🡪 Screen use (γ) | 0.069 | 0.124 | .580 | -0.081 | 0.154 | .599 |
| Screen use 🡪 Depression (β) | 0.094 | 0.114 | .410 | 0.119 | 0.081 | .142 |
| Screen use 🡪 Screen use (δ) | -0.069 | 0.105 | .513 | **-0.544** | **0.157** | **.001** |

NB. rows in bold reflect significant parameter estimates.

Table S.6. Cross-lagged standardized effects (RI-CLPM) between depression and time spent using screens by gender for students in Grade 7 at baseline

|  | Males | | | Females | | |
| --- | --- | --- | --- | --- | --- | --- |
|  | b | SE | *p* | b | SE | *p* |
| Total Screen Time | | | | | | |
| Depression 🡪 Depression (α) | **0.249** | **0.112** | **.026** | **0.550** | **0.172** | **.001** |
| Depression 🡪 Screen use (γ) | 0.068 | 0.089 | .501 | 0.217 | 0.138 | .107 |
| Screen use 🡪 Depression (β) | **0.272** | **0.074** | **.000** | **0.253** | **0.112** | **.024** |
| Screen use 🡪 Screen use (δ) | 0.101 | 0.116 | .383 | **0.530** | **0.132** | **.000** |
| Social Media | | | | | | |
| Depression 🡪 Depression (α) | 0.215 | 0.132 | .103 | **0.489** | **0.169** | **.020** |
| Depression 🡪 Screen use (γ) | 0.228 | 0.119 | .056 | 0.089 | 0.126 | .482 |
| Screen use 🡪 Depression (β) | 0.077 | 0.096 | .422 | 0.025 | 0.122 | .841 |
| Screen use 🡪 Screen use (δ) | -0.144 | 0.128 | .261 | **0.348** | **0.137** | **.011** |
| Gaming | | | | | | |
| Depression 🡪 Depression (α) | **0.279** | **0.118** | **.018** | **0.472** | **0.169** | **.005** |
| Depression 🡪 Screen use (γ) | 0.057 | 0.088 | .518 | 0.081 | 0.078 | .300 |
| Screen use 🡪 Depression (β) | -0.030 | 0.133 | .818 | 0.113 | 0.107 | .289 |
| Screen use 🡪 Screen use (δ) | **0.534** | **0.154** | **.001** | **0.331** | **0.118** | **.005** |
| TV/Passive | | | | | | |
| Depression 🡪 Depression (α) | **0.297** | **0.106** | **.005** | **0.480** | **0.166** | **.004** |
| Depression 🡪 Screen use (γ) | 0.052 | 0.103 | .612 | 0.139 | 0.142 | .328 |
| Screen use 🡪 Depression (β) | -0.110 | 0.081 | .175 | 0.115 | 0.110 | .294 |
| Screen use 🡪 Screen use (δ) | -0.010 | 0.120 | .935 | **0.349** | **0.131** | **.008** |
| Web | | | | | | |
| Depression 🡪 Depression (α) | **0.269** | **0.103** | **.009** | **0.473** | **0.162** | **.004** |
| Depression 🡪 Screen use (γ) | 0.121 | 0.077 | .114 | -0.014 | 0.126 | .912 |
| Screen use 🡪 Depression (β) | 0.107 | 0.086 | .213 | 0.051 | 0.109 | .642 |
| Screen use 🡪 Screen use (δ) | **0.383** | **0.095** | **.000** | **0.349** | **0.130** | **.007** |

NB. rows in bold reflect significant parameter estimates.

Table S.7. Cross-lagged standardized effects (RI-CLPM) between depression and time spent using screens by gender for students in Grade 9 at baseline

|  | Males | | | Females | | |
| --- | --- | --- | --- | --- | --- | --- |
|  | b | SE | *p* | b | SE | *p* |
| Total Screen Time | | | | | | |
| Depression 🡪 Depression (α) | 0.396 | 0.207 | .055 | -0.115 | 0.258 | .656 |
| Depression 🡪 Screen use (γ) | -0.009 | 0.159 | .955 | 0.026 | 0.146 | .857 |
| Screen use 🡪 Depression (β) | 0.107 | 0.165 | .518 | 0.187 | 0.181 | .302 |
| Screen use 🡪 Screen use (δ) | **0.283** | **0.142** | **.046** | **0.326** | **0.161** | **.044** |
| Social Media | | | | | | |
| Depression 🡪 Depression (α) | **0.486** | **0.169** | **.004** | -0.164 | 0.163 | .315 |
| Depression 🡪 Screen use (γ) | **0.143** | **0.067** | **.033** | -0.087 | 0.063 | .167 |
| Screen use 🡪 Depression (β) | 0.401 | 0.219 | .067 | -0.131 | 0.258 | .610 |
| Screen use 🡪 Screen use (δ) | **0.514** | **0.090** | **.000** | 0.230 | 0.146 | .115 |
| Gaming | | | | | | |
| Depression 🡪 Depression (α) | 0.319 | 0.222 | .151 | -0.098 | 0.210 | .639 |
| Depression 🡪 Screen use (γ) | 0.192 | 0.130 | .140 | 0.028 | 0.117 | .812 |
| Screen use 🡪 Depression (β) | 0.031 | 0.131 | .814 | 0.271 | 0.170 | .111 |
| Screen use 🡪 Screen use (δ) | 0.166 | 0.090 | .066 | **0.460** | **0.145** | **.001** |
| TV/Passive | | | | | | |
| Depression 🡪 Depression (α) | 0.008 | 0.254 | .976 | -0.145 | 0.266 | .584 |
| Depression 🡪 Screen use (γ) | 0.067 | 0.117 | .565 | -0.085 | 0.121 | .483 |
| Screen use 🡪 Depression (β) | 0.131 | 0.171 | .446 | -0.013 | 0.143 | .929 |
| Screen use 🡪 Screen use (δ) | **0.383** | **0.099** | **.000** | 0.105 | 0.107 | .321 |
| Web | | | | | | |
| Depression 🡪 Depression (α) | 0.138 | 0.230 | .549 | 0.008 | 0.224 | .971 |
| Depression 🡪 Screen use (γ) | 0.075 | 0.110 | .497 | 0.061 | 0.118 | .607 |
| Screen use 🡪 Depression (β) | 0.048 | 0.140 | .732 | 0.187 | 0.137 | .173 |
| Screen use 🡪 Screen use (δ) | **0.320** | **0.095** | **.001** | 0.228 | 0.141 | .105 |

NB. rows in bold reflect significant parameter estimates.

Table S.8: Sensitivity analysis - Cross-lagged standardized effects (RI-CLPM) between depression and time spent using screens using screen use at wave 2, 4, and 6 only, instead of using average times of 1+2, 3+4, 5+6

|  | b | SE | *p* | Model fit |
| --- | --- | --- | --- | --- |
| Total Screen Time | | | | |
| Depression 🡪 Depression (α) | **0.316** | **0.051** | **.000** | RMSEA = 0.056 χ^2^ = 64.1  *p* < .001  ICC_Depression_ = 0.32  ICC_Screen Time_ = 0.44 |
| Depression 🡪 Screen use (γ) | **0.162** | **0.042** | **.000** |  |
| Screen use 🡪 Depression (β) | **0.094** | **0.037** | **.011** |  |
| Screen use 🡪 Screen use (δ) | **0.124** | **0.044** | **.005** |  |
| Social Media | | | | |
| Depression 🡪 Depression (α) | **0.315** | **0.045** | **.000** | RMSEA = 0.068 χ^2^ = 89.2  p < 0.001  ICC_Depression_ = 0.33  ICC_Social media_ = 0.34 |
| Depression 🡪 Screen use (γ) | 0.045 | 0.048 | .355 |  |
| Screen use 🡪 Depression (β) | 0.013 | 0.044 | .765 |  |
| Screen use 🡪 Screen use (δ) | **0.274** | **0.049** | **.000** |  |
| Gaming | | | | |
| Depression 🡪 Depression (α) | **0.309** | **0.049** | **.000** | RMSEA = 0.063 χ^2^ = 77.7  *p* < .001  ICC_Depression_ = 0.33  ICC_Gaming_ = 0.34 |
| Depression 🡪 Screen use (γ) | 0.065 | 0.039 | .094 |  |
| Screen use 🡪 Depression (β) | -0.001 | 0.036 | .978 |  |
| Screen use 🡪 Screen use (δ) | **0.160** | **0.038** | **.000** |  |
| TV/Passive | | | | |
| Depression 🡪 Depression (α) | **0.299** | **0.046** | **.000** | RMSEA = 0.032 χ^2^ = 28.5  *p* = .005  ICC_Depression_ = 0.36  ICC_TV_ = 0.42 |
| Depression 🡪 Screen use (γ) | 0.083 | 0.046 | .072 |  |
| Screen use 🡪 Depression (β) | 0.060 | 0.037 | .104 |  |
| Screen use 🡪 Screen use (δ) | **0.110** | **0.045** | **.014** |  |
| Web | | | | |
| Depression 🡪 Depression (α) | **0.311** | **0.046** | **.000** | RMSEA = 0.031 χ^2^ = 25.5  *p* = .008  ICC_Depression_ = 0.33  ICC_Web_ = 0.27 |
| Depression 🡪 Screen use (γ) | 0.043 | 0.043 | .321 |  |
| Screen use 🡪 Depression (β) | 0.050 | 0.038 | .185 |  |
| Screen use 🡪 Screen use (δ) | **0.188** | **0.042** | **.000** |  |

NB. rows in bold reflect significant parameter estimates.

Table S.9. Sensitivity analysis: Cross-lagged standardized effects (RI-CLPM) between depression and time spent using screens by gender where co-efficients between T1 and T2 and between T2 and T3 are not constrained to be equal

|  | T1 - T2 | | | T2 - T3 | | |
| --- | --- | --- | --- | --- | --- | --- |
|  | b | SE | *p* | b | SE | *p* |
| Total Screen Time | | | | | | |
| Depression 🡪 Depression (α) | **0.256** | **0.072** | **< .001** | **0.190** | **0.062** | **.002** |
| Depression 🡪 Screen use (γ) | **0.138** | **0.060** | **.022** | **0.156** | **0.061** | **.010** |
| Screen use 🡪 Depression (β) | **0.124** | **0.054** | **.021** | **0.139** | **0.061** | **.022** |
| Screen use 🡪 Screen use (δ) | **0.376** | **0.065** | **< .001** | **0.310** | **0.062** | **< .001** |

NB. rows in bold reflect significant parameter estimates.
